# HIF prolyl hydroxylase inhibition protects skeletal muscle from eccentric contraction-induced injury

**DOI:** 10.1186/s13395-018-0179-5

**Published:** 2018-11-13

**Authors:** Andrew N. Billin, Samuel E. Honeycutt, Alan V. McDougal, Jaclyn P. Kerr, Zhe Chen, Johannes M. Freudenberg, Deepak K. Rajpal, Guizhen Luo, Henning Fritz Kramer, Robert S. Geske, Frank Fang, Bert Yao, Richard V. Clark, John Lepore, Alex Cobitz, Ram Miller, Kazunori Nosaka, Aaron C. Hinken, Alan J. Russell

**Affiliations:** 1 Muscle Metabolism Discovery Performance Unit, GlaxoSmithKline, King of Prussia, PA USA; 2 Metabolic Pathways and Cardiovascular Therapy Area, GlaxoSmithKline, King of Prussia, PA USA; 3 Target Sciences, GlaxoSmithKline, King of Prussia, PA USA; 4 Clinical Statistics, GlaxoSmithKline, King of Prussia, PA USA; 5 School of Medical and Health Sciences, Edith Cowan University, Joondalup, WA Australia

**Keywords:** Eccentric injury, HIF activation, Skeletal muscle, Prolyl hydroxylase, Protection

## Abstract

**Background:**

In muscular dystrophy and old age, skeletal muscle repair is compromised leading to fibrosis and fatty tissue accumulation. Therefore, therapies that protect skeletal muscle or enhance repair would be valuable medical treatments. Hypoxia-inducible factors (HIFs) regulate gene transcription under conditions of low oxygen, and HIF target genes EPO and VEGF have been associated with muscle protection and repair. We tested the importance of HIF activation following skeletal muscle injury, in both a murine model and human volunteers, using prolyl hydroxylase inhibitors that stabilize and activate HIF.

**Methods:**

Using a mouse eccentric limb injury model, we characterized the protective effects of prolyl hydroxylase inhibitor, GSK1120360A. We then extended these studies to examine the impact of EPO modulation and infiltrating immune cell populations on muscle protection. Finally, we extended this study with an experimental medicine approach using eccentric arm exercise in untrained volunteers to measure the muscle-protective effects of a clinical prolyl hydroxylase inhibitor, daprodustat.

**Results:**

GSK1120360A dramatically prevented functional deficits and histological damage, while accelerating recovery after eccentric limb injury in mice. Surprisingly, this effect was independent of EPO, but required myeloid HIF1α-mediated iNOS activity. Treatment of healthy human volunteers with high-dose daprodustat reduced accumulation of circulating damage markers following eccentric arm exercise, although we did not observe any diminution of functional deficits with compound treatment.

**Conclusion:**

The results of these experiments highlight a novel skeletal muscle protective effect of prolyl hydroxylase inhibition via HIF-mediated expression of iNOS in macrophages. Partial recapitulation of these findings in healthy volunteers suggests elements of consistent pharmacology compared to responses in mice although there are clear differences between these two systems.

**Electronic supplementary material:**

The online version of this article (10.1186/s13395-018-0179-5) contains supplementary material, which is available to authorized users.

## Introduction

Under conditions of trauma or extreme physical exercise, skeletal muscle damage occurs and sets off an orchestrated series of events to replace and repair damaged tissue [1]. The dynamics of this process can be disrupted by chronic inflammation and age, resulting in inefficient replacement of muscle and the accumulation of connective tissue and fat [2, 3].

The transcription factor hypoxia-induced factor 1α (HIF1α) is a primary regulator of the homeostatic response to hypoxia [2], and several lines of evidence point to the importance of hypoxic response to skeletal muscle repair. Both HIF1α and the HIF-target gene, VEGF, are elevated by strenuous exercise [4, 5], and VEGF stimulates muscle angiogenesis and myoblast proliferation [6]. Another HIF-target gene, erythropoietin (EPO), also stimulates myoblast proliferation and muscle recovery after injury. One of the primary mediators of HIF1α activity in muscle is local oxygen concentration, which can drop significantly during exercise or injury [7, 8]. Under normoxic conditions, oxygen-regulated HIF proteins (primarily HIF1α, HIF2α) are hydroxylated and targeted for proteasomal degradation by HIF-prolyl 4-hydroxylases (PHD1,2,3) which require molecular O_2_ for activity. Under low oxygen tension, PHD activity is reduced, stabilizing HIF and allowing transcription of target genes [9]. Recently, small molecule inhibitors of PHD proteins have been identified that stabilize HIF in vivo [10]. We took advantage of these pharmacologic tools to test the hypothesis that acute HIF activation would protect against muscle damage induced by lengthening (eccentric) muscle contractions and accelerate muscle repair in both mice and humans.

## Results

**Fig. 1:**
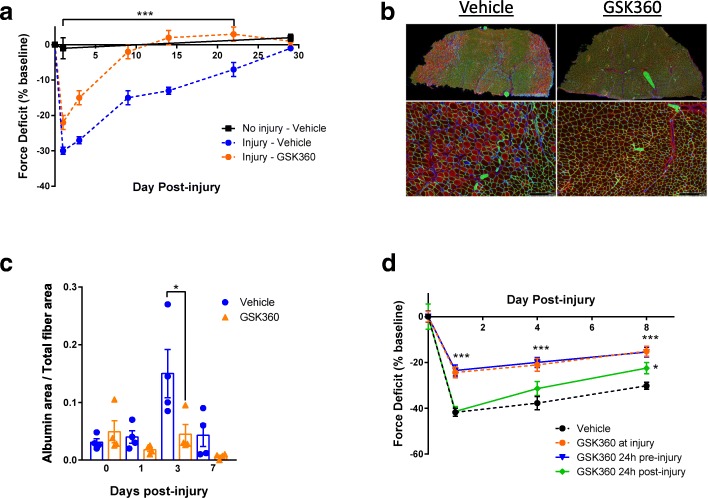
Prolyl hydroxylase inhibitors protect skeletal muscle from exercise-induced injury. **a** Maximal limb force in mice before and after eccentric muscle exercise. Mice were treated with vehicle or GSK360 daily, starting 10 min after eccentric exercise. Limb force was measured for 28 days. Uninjured limb force included as a control. Data are normalized to pre-exercise force (*n* = 8). **b** Representative images of immunohistochemical staining for albumin (red), laminin (green), and nuclei (blue) in gastrocnemius muscle 3 days after eccentric exercise (*n* = 4/group). Upper panels: low power images of full cross section of gastrocnemius. Lower panels: higher magnifications of the rectangular areas above (scale bars = 250 μM). Arrows: albumin-positive myofibers. Asterisks: albumin-negative fibers. **c** Quantification of albumin-positive fibers measured as a proportion of total fibers (*n* = 4/group). **d** Maximal limb force in mice before and after eccentric muscle damage. Mice were treated daily with vehicle or GSK360 24 h before, at the time of injury, or 24 h after eccentric exercise. Limb force was measures for 8 days (*n* = 8). Data shown as the mean ± SEM. Two-way ANOVA followed by Holm-Sidak’s test corrected for multiple comparisons; *P < 0.05, **P < 0.01, ***P < 0.001 versus vehicle-dosed cohort (unless otherwise indicated)

Skeletal muscle is subject to dramatic changes in length and strain during contraction. One of the transcription factors involved in initiating the tissue response to acute exercise was recently shown to be HIF1α [11]. We asked whether inhibiting PHD enzymes would be beneficial for muscle repair after injury. Using eccentric, lengthening contractions to cause acute muscle damage with subsequent inflammation and tissue remodeling [12] via a non-invasive footplate system in the *gastrocnemius* muscle of mice [13], muscle force was decreased on average by 30–40%, but fully recovered to the initial force after 30 days. In contrast, daily dosage of the PHD inhibitor GSK1120360A (GSK360) [14], starting 10 min after injury, provided a significant protective effect at 24 h and full restoration of muscle force 9 days after damage (Fig. 1a, Additional file 1: Figure S1a). Furthermore, we found that fluorescent albumin incorporation was decreased by 70% 3 days post-injury with GSK360 treatment (Fig. 1b, c), indicating PHD inhibition also resulted in histological evidence of muscle protection.

As we saw a biphasic GSK360 effect on force recovery, we asked whether removing the initial protection from injury would still result in accelerated recovery. Instead of dosing at the time of injury, we delayed the initial dose of GSK360 until 24 h post-eccentric damage. Under these conditions, we found that recovery was still accelerated compared to control animals (Fig. 1d; GSK360 24 h post-injury), indicating that activation of hypoxic signaling both decreased initial injury and accelerated muscle recovery.

**Fig. 2:**
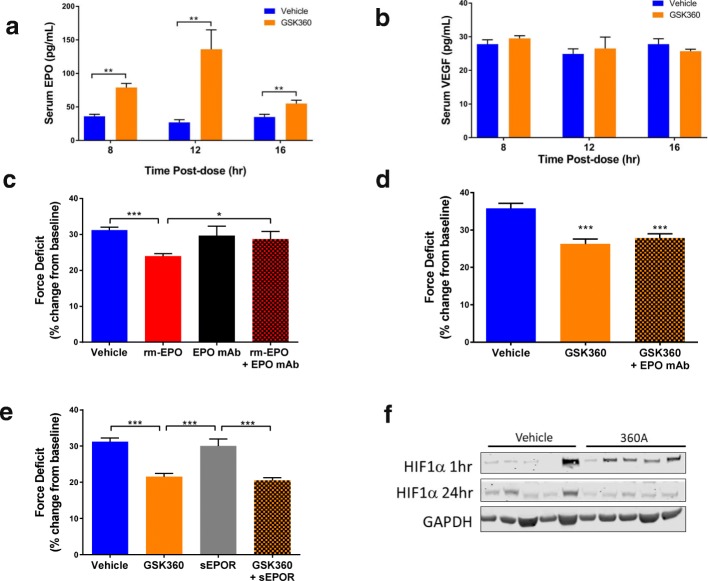
Prolyl hydroxylase inhibitors exert their protective effects independently of EPO. **a** Serum EPO concentration and **b** Serum VEGF concentration after oral administration of 10 mg/kg GSK360 in mice (*n* = 8). **c** Force deficit 24 h after eccentric injury. Mice were treated daily with rEPO, with and without an EPO neutralizing antibody (*n* = 8). **d** Force deficit 24 h after eccentric injury of mice co-treated with EPO neutralizing antibody and GSK360 (*n* = 8). **e** Force deficit 24 h after eccentric injury of mice co-treated with soluble EPO receptor and GSK360 (n = 8). **f** Western blot of HIF1α protein levels from muscle lysates 1 h and 24 h after limb injury. GAPDH used as a loading control

Dosing with GSK360 has been shown to induce EPO production in vivo [14]. As EPO has been previously linked to improved muscle repair after injury [15], we tested whether the elevation of EPO by GSK360 was responsible for its protective effects. At the maximal effective dose (10 mg/kg) of GSK360, we found elevations in circulating EPO, but not VEGF (Fig. 2a, b). We then treated mice daily with recombinant EPO, with or without an EPO neutralizing antibody, beginning 2 days prior to eccentric injury to elucidate the effects of GSK360-induced EPO production on muscle recovery. Recombinant EPO was protective to muscle if administered before injury (Fig. 2c). However, pre-dosing with either an EPO neutralizing antibody, or a soluble EPO receptor decoy [16] prior to GSK360 treatment did not alter GSK360’s protective muscle effects (Fig. 2d, e), while lowering Hct and RBC counts (Additional file 1: Figure S1b, c), suggesting that the protection afforded by GSK360 was not dependent on EPO.

That EPO was not required for muscle protection with GSK360 indicated another mechanism through which PHD inhibition might affect muscle injury, possibly via HIF1α upregulation in muscle itself. HIF1α protein was transiently elevated in compound treated muscle after injury (Fig. 2f), so we surveyed the early transcriptional effects of GSK360 in damaged muscle. Unexpectedly, we found no significant transcriptional changes specific to treatment in muscle (Additional file 2: Figure S2a, Additional file 3: Table S1) and that compound concentration in muscle was low relative to other organs (Additional file 2: Figure S2b), together suggesting another tissue compartment was potentially responsible for the effects on muscle repair.

**Fig. 3:**
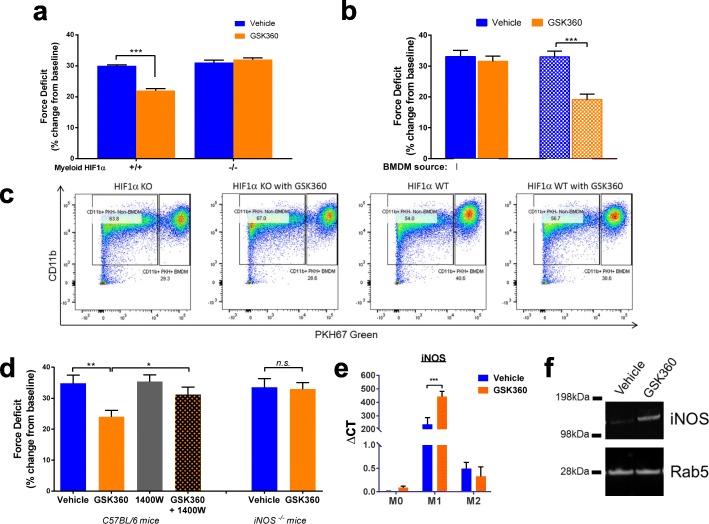
Prolyl hydroxylase inhibitors exert their protective effects via macrophage HIF1α and iNOS. **a** Force deficit 24 h after eccentric injury in indicated strains of myeloid HIF1α KO mice administered vehicle or GSK360 (*n* = 8). **b** Force deficit 24 h after eccentric injury in myeloid HIF1α KO mice with exogenous BMDM (either HIF1α KO or WT) injected intramuscularly following eccentric injury and treated with vehicle or GSK360 (*n* = 8). **c** FACS analysis of PKH67-green labeled myeloid populations recovered from injured muscles 24 h after injection and treatment. **d** Force deficit 24 h after eccentric injury in WT mice treated with vehicle, iNOS inhibitor 1400 W and/or GSK360. Right side; force deficit 24 h after eccentric injury in iNOS KO mice administered vehicle or GSK360 (*n* = 8). **e** iNOS gene expression measured by qPCR. Bone marrow-derived macrophages (BMDM) were cultured and polarized to promote classical M1 or M2 activation or left inactivated (M0) and treated with vehicle or GSK360 for 24 h. **f** iNOS protein expression in M1-polarized macrophages treated with GSK360 for 24 h before protein analysis by Western blot. Rab5 was used as a loading control. All data in the figure are shown as the mean ± SEM. Two-way ANOVA followed by Holm-Sidak’s test corrected for multiple comparisons; *P < 0.05, **P < 0.01, ***P < 0.001 versus the vehicle dosed cohort (unless otherwise indicated)

One possible effector that may play a role in the blunting of damage and acceleration of recovery would be the immune response following muscle damage. Cells of the myeloid lineage dominate muscle infiltrates after damage [17], and myeloid *Hif1a* knockout (HIF1α KO) mice exhibit delayed recovery after muscle trauma [8] although this finding has been recently disputed [18]. Using myeloid HIF1α KO mice, we asked whether altering hypoxic signaling in immune cells would affect recovery from muscle injury. Using the same eccentric damage protocol as before, we found that GSK360 was not protective in either myeloid HIF1α KO homo- or heterozygous mice (Fig. 3a), implicating HIF activation in myeloid cells in the muscle-protective response to GSK360.

Early myeloid infiltration into injured muscle is dominated by neutrophils and macrophages, with macrophages increasing in number and activity by 24 h after acute injury [19] (Additional file 2: Figure S2c). As macrophages play an important role in promoting skeletal muscle protection and repair [20], we asked whether HIF-responsive macrophages, rather than neutrophils, drove the primary GSK360 response in the muscle. To do so, we cultured bone marrow-derived macrophages (BMDMs) from both myeloid HIF1α KO and myeloid HIF1α expressing mice. The plasma membranes of these BMDM were fluorescently labeled with PKH67-green and injected intramuscularly into the gastrocnemius of myeloid HIF1α KO mice immediately following eccentric injury. Adoptive transfer of HIF1α expressing macrophages re-established the protective effect of GSK360 seen in wild-type mice (c.f. Fig. 1), in contrast to those muscles that received HIF1α-deficient BMDM (Fig. 3b). FACS analysis of the myeloid cell population recovered from the gastrocnemius muscles 24 h following injury confirmed that most of the PKH67-green cells were positive for the macrophage marker, CD11b^+^ (Fig. 3c). We conclude that GSK360’s protective effects are mediated through HIF activation and signaling in macrophages.

Macrophages are highly responsive to hypoxic signaling, with HIF1α regulating a number of genes that may contribute to muscle protection including TNFα, IL-1β, iNOS, and VEGF [21]. Our primary focus was on iNOS as both nitric oxide [22] and iNOS itself [23] are regulators of myogenesis and muscle repair. Furthermore, they are highly expressed by classically activated M1 macrophages found to be present within the first 24 h following muscle injury [24], fitting the timeframe in which GSK360 appeared to be working. We first manipulated NO activity with pharmacological tools, co-treating mice with both GSK360 and 1400 W, an iNOS inhibitor [25]. The addition of 1400 W blunted the GSK360 effect on muscle injury (Fig. 3d). We also measured force production after eccentric contractions in an iNOS KO mouse [26]; similarly to inhibiting iNOS, GSK360 failed to protect muscle after eccentric injury (Fig. 3d). Treating BMDM in culture with GSK360 resulted in increased expression of HIF1α only in classically activated M1 macrophages (Additional file 2: Figure S2d), along with a dramatic increase in iNOS expression (Fig. 3e, f). Together, these data point to PHI mediating a protective effect in muscle via activation of HIF1α in macrophages and the subsequent upregulation of iNOS.

**Fig. 4:**
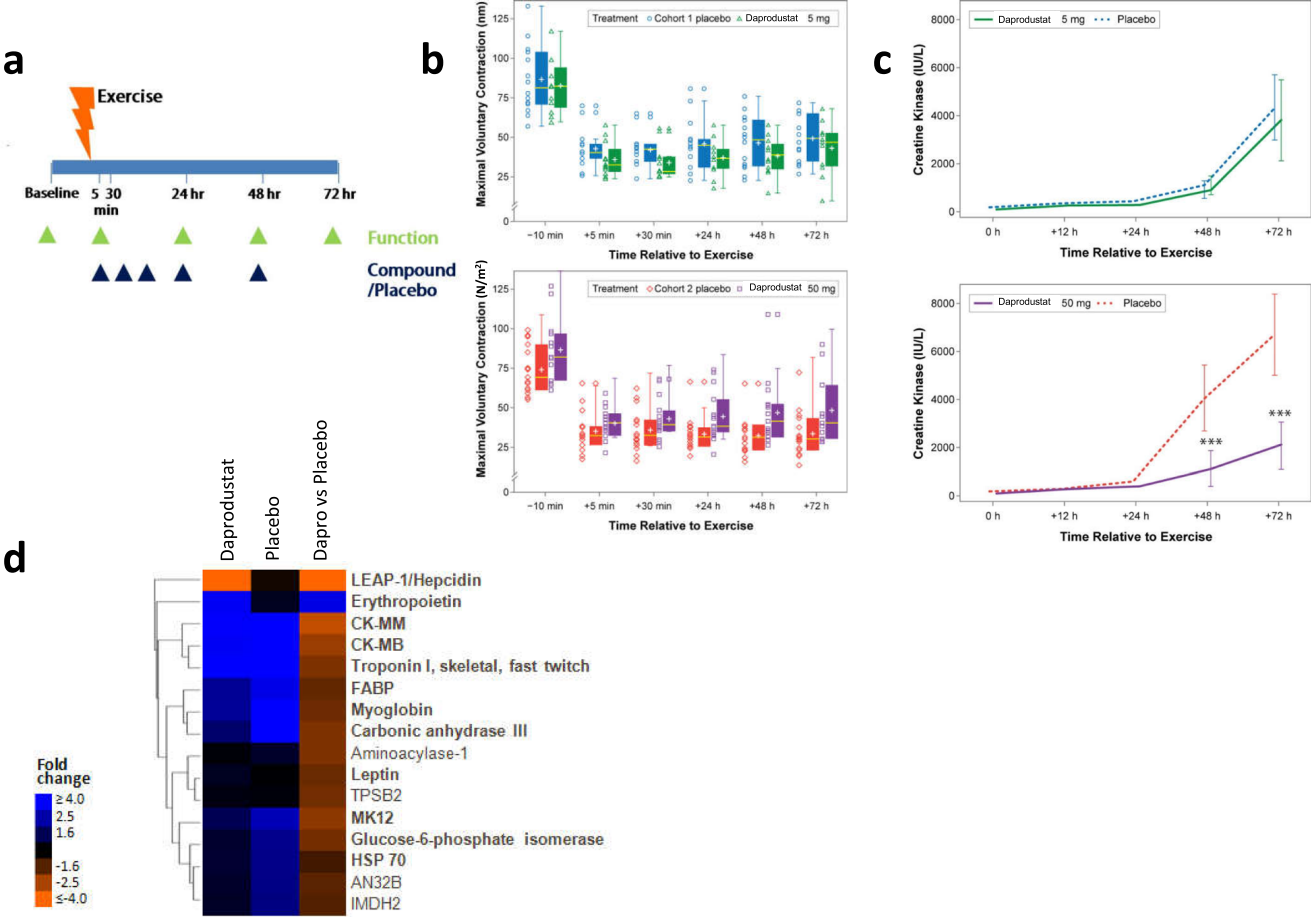
Prolyl hydroxylase inhibitor daprodustat reduces markers of muscle damage in healthy male volunteers after eccentric exercise. **a** Clinical study design. **b** Maximum voluntary force at an arm angle of 90^o^ before and after eccentric exercise in two separate studies. Boxed area represents a 75% range of values; central line indicates the median and the cross the mean value. Individual values are displayed to the left (top figure, placebo *n* = 14, daprodustat 5 mg *n* = 12; bottom figure, placebo *n* = 15, daprodustat 50 mg *n* = 15). **c** Serum creatine kinase before and after eccentric exercise in the two separate studies. **d** Heatmap and hierarchical clustering of the top 16 serum proteins changed between pre-exercise and 3 days post exercise and the effect of 50 mg daprodustat on their levels. The first two columns display fold changes pre- vs. postexercise for daprodustat-treated and placebo-treated participants, respectively. The third column shows daprodustat fold change treated vs. placebo-treated samples postexercise. Protein names in bold denote a significant association with muscle-related phenotypes and/or pathways (each group *n* = 15). All data in the figure are shown as the mean ± SEM. Repeat measure ANOVA for multiple comparisons; ***P < 0.001)

In humans, performing unaccustomed eccentric exercise also leads to tissue damage, functional deficits, and muscle pain often referred to as delayed onset muscle soreness [27]. We took advantage of this to test the ability of a clinical stage PHI, daprodustat [28], to reduce muscle damage in healthy volunteers subjected to eccentric exercise of the elbow flexors in a double-blind placebo-controlled study. Daprodustat is structurally related to GSK360 and in preclinical studies also protected mice from functional deficits after eccentric injury (Additional file 4: Figure S3a). Subjects performed 30–60 contractions of their non-dominant arm to induce more than 40% strength loss immediately post-exercise and then were randomized to receive a total of five doses of either placebo or daprodustat (Fig. 4a). Arm function was measured as maximum voluntary contraction (MVC) force generated at a fixed elbow joint angle of 90^o^ and was recorded 10 min pre-exercise and 5 min, 30 min, 24 h, 48 h, and 72 h post-exercise. We also measured range of motion and serum muscle proteins over 3 days. The study was arranged in two separate cohorts of placebo vs 5 mg daprodustat, and placebo vs 50 mg daprodustat (Additional file 5: Table S2 describes demographic data). There were 15 total adverse effects; none serious or thought to be compound-related, with the most frequent being exercise-induced arm muscle pain (myalgia, Additional file 6: Table S3). Both MVC force and range of motion were reduced by exercise but were not altered with either dose of daprodustat (Fig. 4b, Additional file 4: Figure S3b). However, increases in serum creatine kinase activity after exercise were significantly lower in individuals administered 50 mg daprodustat (Fig. 4c).

We pursued an aptamer-based proteomics approach to identify additional circulating markers of muscle injury and the effects of daprodustat treatment in serum from the placebo and 50 mg daprodustat groups (Somalogic, Inc.). Comparison of pre- and post-injury serum in the placebo group revealed that 9 of 11 proteins increased with exercise were associated with skeletal muscle and were all lowered by daprodustat (Fig. 4d, Additional file 7: Table S4). These changes are consistent with the mouse data, suggesting a reduction in tissue damage after muscle injury with HIF prolyl hydroxylase inhibition.

## Discussion

PHD regulation of HIF1α has been implicated in the protection of the heart, brain, and liver after an ischemic insult [14, 29, 30]. The mechanism of this protection appears to vary by tissue type and often reflects the effect of chronic HIF1α activation, robust target gene activation, and tissue remodeling after PHD protein knockout. Compared to these reports, we were surprised by both the acute nature of the skeletal muscle protective effect and its modest transcriptional response in muscle in our preclinical system. Higher doses (30 mg/kg) of GSK360 have been associated with HIF1α target gene activation in muscle [14], but our studies identified a more sensitive myeloid population that drives muscle protection in an iNOS-dependent manner without large scale transcriptional responses. iNOS is required for the efficient recovery of skeletal muscle after injury in mice [23], but to our knowledge, this is the first time that iNOS has been implicated in an acute protective role after muscle injury. In skeletal muscle ischemia-reperfusion injury, iNOS is generally associated with a delayed inflammatory response and leads to high NO levels, membrane lysis, and skeletal muscle breakdown [31]. A complex balance exists between protective and detrimental effects of NO dependent upon the time and extent of NO release [32]. Therefore, we presume that the modest inflammatory response and NO generation associated with eccentric contraction-induced muscle injury leads to a greater protective role for NO generated through iNOS. We demonstrate that the macrophage HIF1α compartment is essential for the protective effect of PHD inhibition, which is consistent with their presumed role in membrane repair after eccentric contraction-induced muscle injury [20]. The exact mechanism by which the hypoxic response generated by PHD inhibition in macrophages is driving the protection and recovery from the muscle injury is as yet undetermined, but it is likely to either be remodeling the inflammatory response or accelerating satellite cell activation [33]. GSK360 and daprodustat are both non-selective inhibitors of PHD1, 2, and 3 [14, 34], and polarized macrophages express all three PHD isoforms [35], making determination of which isoform(s) are required for muscle protection difficult without more detailed studies. In preliminary experiments, GSK360 treatment of M1/M2 polarized BMDM cells resulted in the upregulation of both inducible PHDs (PHD2 and PHD3) in M1 but not M2 macrophages (data not shown). This would suggest reflex compensation from elevated HIF1α in M1 cells but does not suggest which key PHD(s) are driving this response.

While healthy volunteer data did not recapitulate the functional protection of PHI seen in mice, circulating markers of muscle damage were reduced by daprodustat in the highest dose group. This is possibly due to differences between these two systems. The cause of voluntary strength deficits in people after eccentric exercise are not well understood [36] and are associated with modest changes in histological and inflammatory markers of muscle damage [37, 38] compared to the more robust damage we observed in mouse models. Force deficits in people can also occur without elevation of circulating markers of muscle damage after preconditioning exercise [39], suggesting that mechanisms independent of histological damage, such as excitation-contraction coupling failure [36], may be the determinants of human functional deficits. Further, voluntary muscle contraction in humans removes the contribution of factors such as muscle pain to reductions in arm strength, which are not captured by involuntary electrical stimulation in the mouse. We also note that the volunteers used in this study were somewhat overweight (mean BMI 26–28.5), possibly due to the entry requirement for no regular exercise, a factor that could also influence the outcome of the study.

Notably, the circulating signature of muscle-related proteins elevated by exercise and decreased by daprodustat treatment in human volunteers is remarkably similar to that observed in muscular dystrophy patients [40]. This likely reflects similarities between muscle injury after eccentric exercise in healthy individuals and muscle injury due to structural fragility in dystrophic patients. Further research is underway to understand if muscle protection through PHD inhibition may provide protection in the background of muscular dystrophy.

## Conclusion

We have uncovered a novel connection between HIF1α and iNOS that contributes to muscle protection after exercise-driven injury in mice. Evidence suggests that some elements of this protective effect may also be competent in healthy volunteers after eccentric injury, although there are clear differences in the magnitude of response between mouse and man. Our data implicates the HIF signaling axis as an important modulator of inflammatory activity during skeletal muscle repair and injury and further indicates that this mechanism may be of therapeutic importance in the treatment of acute muscle injury.

## Materials and methods

### Preclinical methods

#### Animals

Myeloid cell-specific *Hif1a* null (HIF1α KO) (B6.129-Hif1atm3Rsjo/J x B6.129P2-Lyz2tm1(cre)Ifo/J), iNOS KO (B6.129P2-iNOStmLau/J), and normal C57Bl6/NJ mice were purchased from Jackson labs. Male adult mice (3–4 months-old) were housed individually (12 h:12 h day:night cycle) in a temperature- and humidity-controlled environment (73.5 ± 5.5 °F and 50 ± 20%, respectively) and fed standard chow (LabDiet 5001, Purina Mills, LLC, St. Louis, MO) and water ad libitum. Mice were utilized for experimentation following a 7-day acclimation period and randomized into treatment groups by body weight. All studies were conducted in accordance with the GSK Policy on the Care, Welfare and Treatment of Laboratory Animals. All protocols were reviewed and approved by the Institutional Animal Care and Use Committee of GSK.

#### In vivo reagents

GSK1120360 (GSK360) was formulated in 0.5% hydroxypropylmethylcellulose (Acros Organics, Lot#A0320015, Code 244020010, CAS 9004-65-3) and 0.1% Tween80 (Fisher, Lot#141362, Cat# T164-500) (HPMC:Tween) vehicle and administered (PO) daily at specified doses beginning on the day of eccentric damage (with the exception of the experiment in Fig. 1b, with timings as indicated). The iNOS-specific inhibitor 1400 W (20 mg/kg) and recombinant mouse erythropoietin (EPO) (20 μg/kg) were injected subcutaneously in saline-based vehicles. 1400 W was administered 2-h prior to eccentric damage, while EPO was dosed daily for 3 days leading up to the day of damage. For EPO neutralization, 600 μg of a monoclonal antibody targeting EPO (R&D Systems, MAB959) was injected SC at days − 3 and 1 prior to eccentric damage. For EPO depletion studies, 200 μg/kg of a soluble EPO receptor (R&D Systems, 1390-ER-050) was injected SC twice a day for 3 days prior to eccentric damage.

#### In vivo muscle contractility

Mice were placed on a warming plate (30–32 °C) and anesthetized using 2%/L O_2_ isoflurane. Right hind limbs were first shaved and then pinned at the knee at 90° with foot strapped into a mouse “shoe” which doubles as a motor arm and force transducer (Aurora Scientific Instruments 305C, Aurora). Platinum sub-dermal electrodes (Grass Instruments; West Warwick, RI) were inserted dorsally and ventrally to the femur for field stimulation of the sciatic nerve. Because the mouse gastrocnemius, plantaris, and soleus muscles are larger and stronger as a complex than the ankle dorsiflexors (TA and EDL), hind limbs produced a net plantarflexion response to sciatic activation. Tetanic stimuli of 150 Hz at 200 μs pulse width (with 2 mA current and 15 V) for 0.8 s were recorded as maximal isometric limb forces while single 200 μs pulses were used to elicit maximal twitch force values. Muscle contractility was evaluated immediately prior to injury (baseline force day 0), day 1 post-injury, and then at varying longitudinal points thereafter (as indicated).

#### Eccentric contraction-induced muscle injury

To induce muscle injury, mice were positioned as described above and initial baseline maximal twitch and tetanic limb force measurement were performed. After a 2-min rest interval, mice underwent a unilateral eccentric muscle contraction protocol in which the hind limb plantarflexor muscles were forcibly lengthened using the automated footplate (30° angular rotation, 1800°/s) while being concomitantly stimulated to contract (150 Hz; 200 μs pulse width; 2 mA and 15 V). A bout of eccentric exercise consisted of 1 forced lengthening contraction every 10 s for a total of 60 repetitions.

#### Histology

With the muscle still in situ, an ink mark was made on the muscle belly to serve as a guide for reproducible collection of sections for analysis. Following excision, the gastrocnemius muscles were oriented to provide cross sectional fiber profiles when sectioned. A small drop of OCT media (Sakura Finetek, USA) was placed on a cork circle and the muscle was stabilized by placing the distal end of the muscle into the media. The muscle was maintained in a vertical position while immersing it into liquid nitrogen cooled isopentane for 10 to 15 s until frozen; samples were held at − 80 °C until sectioned. Ten-micrometer frozen sections were prepared in a cryostat and mounted on glass slides. Tissue sections were fixed with 4% paraformaldehyde diluted in PBS for 5 min at room temperature immediately prior to immunohistochemistry (IHC). Immunostaining was carried out on an automated system (Ventana Medical, USA). Briefly, the sections were blocked with 10% normal goat serum for 32 min at 37 °C prior to application of a cocktail of anti-laminin and anti-albumin antibodies. Anti-laminin (Ab44941, Abcam, USA) and anti-albumin antibody (Ab19196, Abcam, USA) were applied at 5 μg/ml and 1:10000 respectively for 1 h at 37 °C. Albumin and laminin were detected using species-specific fluorophore conjugated secondary antibodies. Albumin immunoreactivity was demonstrated using an Alexa594 conjugate (A-11037, Invitrogen, USA) at 5 μg/ml and laminin expression using an Alexa488 conjugate (A-11006, Invitrogen, USA) at 10 μg/ml; incubation in detection antibodies was for 45 min at 37 °C. Following automated wash steps the nuclei were stained using DAPI (D-3571, Invitrogen, USA) then cover slipped using an aqueous mounting media (P36961, Invitrogen, USA). Stained sections were imaged at × 20 magnification on the Zeiss AxioScan.

#### Quantitative image analysis

A bespoke image analysis algorithim was developed (Definiens, Inc., USA) for use with Definiens Architect XD (Definiens AG, Germany) software. The entire cross-sectional area of the scanned gastronemius muscle was included in the analysis using the above software. Laminin expression was used to isolate individual cross-sectional myofibers. These individual fibers were assessed for their expression of albumin (Optical Density) as well as additional morphometric parameters (e.g., perimeter, area, roundness, myonuclei count and intracellular distribution). Individual fiber data were exported to CSV files for statistical analysis.

#### Gene expression analyses

Mouse gastrocnemius samples were collected and flash frozen in liquid nitrogen then stored at − 80 °C. For RNA isolation, ~ 100 mg samples were used in 1 ml Trizol and isolation was done according to the manufacturers protocol (Ambion Cat #15596-068). Purified RNA went through Qiagen RNA clean up kit (Cat #74204) and eluted in 50 μl elution buffer. For each sample 2 μg total RNA was submitted to Q2 Solutions for Affymetrix analysis, using Affymetrix Gene Chip Mouse Genome 430A 2.0 Array. Raw data files (CEL files) were then pre-processed using the RMA (Robust Multi-chip Average) pipeline [41] in combination with the most current re-annotated probeset definitions [42]. To determine differential mRNA expression, a linear model was fit [43, 44]. The false discovery rate (FDR) was computed as an adjusted *P* value [45] to account for multiple testing and a cut-off of 10% FDR was used to define differential expression. For BMDM work, RNA was isolated from BMDM using a Turbo Capture plate and kit (Qiagen) per manufacturer’s protocol. iNOS expression was quantitated by Taqman qRT-PCR using. Housekeeping genes, Ppia (PRIMERS) and Rpl-p0 (PRIMERS), were used to normalize expression. Data are presented as ΔCt.

#### Plasma assessment of mouse erythropoietin

Plasma EPO and VEGF concentrations were measured using mouse/rat EPO/VEGF serum/plasma multiplexed immunoassay (Meso Scale Discovery, Gaithersburg, MD) according to manufacturers’ protocols.

#### Bone marrow-derived macrophage generation

Tibia and femurs from wild type mice were stripped of muscle tissue and the marrow flushed using a 24-G needle and syringe filled with basal culture media (RPMI 1640 + Glutamax (Gibco), 10% fetal bovine serum (Gibco), 5% horse serum (Gibco), and 1% antibiotic/antimycotic (Gibco)). Isolated marrow cells were gently triturated to a uniform suspension and then filtered through a 40-μm cell strainer. The cell suspension was then centrifuged (500*g* for 5 min) and the resultant pellet resuspended in 1× Red Blood Cell Lysis Buffer (Miltenyi Biotec) and gently agitated for 3 min to lyse red blood cells. The cell suspension was centrifuged, and the resultant pellet resuspended in basal culture media to a final cell concentration of 10^7^ cells/ml. Cells were cultured at an initial density of 1.5 × 10^6^ in 10 cm bacteriological dishes in basal culture media supplemented with 10 ng/ml macrophage colony stimulating factor (MCSF; R&D Systems). At day 4 post-plating, cells were supplemented with basal culture media + 10 ng/ml MCSF. BMDMs were polarized and treated with GSK360 on day 7. To generate M1 macrophages, basal culture media was supplemented with 20 ng/ml recombinant mouse IFNγ (Millipore, cat #407320) + 100 ng/ml of lipopolysaccahride (LPS; SigmaAldrich, cat# L5293). For M2 activation, basal culture media was supplemented with 20 ng/ml recombinant mouse IL-4 (R&D Systems, cat# 404-ML). For naïve, M0 macrophages, only basal culture media was used. GSK360 was then added to designated cultures at a final concentration of 10 μM, with DMSO added as a vehicle control. BMDM were cultured for a final 24 h at 37 °C then isolated by gently pipetting using ice cold PBS (Gibco, pH 7.4) following incubation on ice for 10 min. The suspension was then centrifuged, and the resultant cell pellets processed for either RT-PCR or Western blotting.

#### Adoptive transfer of BMDM

Bone marrow from the tibia and femurs of myeloid HIF1α KO and HIF1α WT littermates were used to culture bone marrow-derived macrophages (BMDMs) as described above. Seven days following initial culture, BMDMs were lifted from culture dishes with Accutase (Gibco). Cell suspensions were pooled in 50-ml conical tubes, centrifuged (500*g* for 5 min), resuspended in serum-free RPMI 1640, counted for cell density numbers, and centrifuged again to pellet. BMDMs were then labeled with PKH67 Green according to manufacturer’s protocol (Sigma-Aldrich). Once labeled, BMDMs were rinsed three times with sterile saline and resuspended at two million cells per 100 μl.

Myeloid HIF1α KO recipient animals were randomized by body weight and initial tetanic force production. Animals were anesthetized, and the eccentric exercise protocol was performed as described above. Immediately following the exercise protocol, 100 μl of BMDM suspension (either HIF1α KO or HIF1α WT) were introduced directly to the injured gastrocnemius via intramuscular injection. Animals were then treated with either vehicle or GSK360 (*N* = 8 for each group). At 24 h post-eccentric damage, force production was assessed as described above.

#### FACS analysis

Muscle tissues were minced and treated with LiberaseTM (Roche) and Dnase I for 2 h at 37 °C. After digestion, tissue homogenates were filtered through 70 μm strainers for cell suspension. For flow cytometry assay, cells were first treated with Fc Block (1:1000; BioLegend), followed by staining surface antigens with UV viability dye (Zombie 1:400; BioLegend), CD11b-PE (M1/70, 1:400), Ly6C-PerCP/Cy5.5 (HK1.4, 1:400), CD45-PE/Cy7 (30-F11, 1:1000) and I-A/I-E (M5/114.15.2, 1:400). Macrophages were labeled with F4/80 mAb (45-4801-80, ThermoFisher). Data were acquired on FACS Aria II and analyzed by FlowJo V10.2.

#### Western blotting

BMDM cell pellets were resuspended in T-PER lysis buffer (Life Technologies) supplemented with Halt™ protease inhibitor cocktail and EDTA (Life Technologies). Suspensions were incubated on ice for 20 min with periodic vortexing, then centrifuged to clarify. Supernatants were assayed for protein content using a microplate BCA assay (ThermoFisher) per manufacturer’s protocol. For each sample, a total of 10 μg of protein were separated on a 4–12% Bis-Tris Bolt™ SDS-PAGE gels (Life Technologies) and transferred to nitrocellulose membranes using an iBlot2 (Life Technologies).

Gastrocnemius muscles were dissected and snap frozen. Approximately 50 mg of frozen tissue was lysed in T-PER lysis buffer (Life Technologies) supplemented with Halt™ protease inhibitor cocktail and EDTA (Life Technologies) using an MPBio Bead Blender. Lysates were incubated on ice for 20 min and then centrifuged to clarify. Supernatants were assayed for protein content using a microplate BCA assay (ThermoFisher) per manufacturer’s protocol. For each sample, 50 μg of protein was separated on 4–12% Bis-Tris Bolt™ SDS-PAGE gels (Life Technologies) and transferred to nitrocellulose membranes using an iBlot2 (Life Technologies). The same sham samples were run on each blot for both 1 h and 24 h time points for comparison to baseline.

Membranes were blocked using Superblock TBS + Tween-20 (ThermoFisher) for 1 h at room temperature. Primary antibodies, diluted in Superblock TBS + Tween-20, were added to the membranes and incubated overnight at 4 °C on a platform rocker. Membranes were washed three times for 15 min at room temperature with TBS + 0.5% Tween-20 (ThermoFisher). Secondary antibodies, diluted in Superblock TBS + Tween-20, were then added to the membranes and incubated for 4 h at room temperature, protected from light. Membranes were then washed as before and imaged on an Odyssey CLx (Li-Cor Systems). Mouse anti-iNOS (BD Transduction Labs, clone 6/iNOS) used at a dilution of 1:1000; rabbit anti-Rab5 (Cell Signaling Technologies) used at a dilution of 1:1000; goat anti-HIF1a (R&D Systems) used at a dilution of 1:1000; donkey anti-mouse 680RD secondary antibody (Li-Cor Biosystems) used at a dilution of 1:10,000; donkey anti-rabbit 800CW (Li-Cor Biosystems) used at a dilution of 1:10,000; donkey anti-goat 800CW (Li-Cor Biosystems) used at a dilution of 1:10,000.

#### HIF1α ELISA

BMDMs polarized to M0, M1, or M2 (as described above) were treated with GSK360 for 24 h (*n* = 2 per dose point). Cells were then lysed using T-PER lysis buffer (Life Technologies) supplemented with Halt™ protease inhibitor cocktail and EDTA (Life Technologies). Resultant suspensions were incubated on ice for 20 min with periodic vortexing then centrifuged to clarify. Supernatants were assayed for protein content using a microplate BCA assay (ThermoFisher) per manufacturer’s protocol. Samples were run in triplicate in a DuoSet HIF1α ELISA (R&D Systems), following the standard protocol. Data was captured with a SpectraMax spectrophotometer and analyzed using GraphPad Prism 7.0.

#### Preclinical statistical analysis

For each experiment, the means and SEM of the parameters measured were determined. Statistical analyses were performed using Student’s *t* test in single-factorial designs. For multifactorial study designs, one- or two-way ANOVA was used as appropriate. Tukey’s (for one-way ANOVA) or Bonferroni (for two-way ANOVA) post hoc test was applied when significant differences were found. Testing for normal variance was performed and confirmed. For mouse experiments, the number of mice per group required to detect biologically significant effect sizes was calculated using an appropriate statistical sample-size formula and indicated in the biometrical planning section of the animal license submitted to the governing authority. Blinding was not done during animal group allocation but was done for some measurements made in the study (i.e., histology). No specific exclusion criteria were applied, as inbred strains, which display uniform phenotypic characteristics, were used exclusively. Analyses were carried out with SigmaPlot v.12 software (Systat Software GmbH, Erkrath) or GraphPad Prism software (GraphPad Software, San Diego). *P* < 0.05 was considered statistically significant.

### Clinical methods

#### Eccentric exercise-induced muscle damage in healthy volunteers

This was a repeat dose, two-cohort, randomized, placebo-controlled, parallel group study in healthy volunteers (Clinicaltrials.gov reference NCT02231190). In each cohort, approximately 30 subjects were enrolled and randomized into a 1:1 ratio between daprodustat and placebo groups (study CONSORT diagram, Additional file 4: Figure S3). The study protocol, any amendments, the informed consent, and other information that required pre-approval were reviewed and approved by an investigational center institutional review board, in accordance with the International Conference on Harmonization of Technical Requirements for Registration of Pharmaceuticals for Human Use (ICH) Good Clinical Practice (GCP) and applicable country-specific requirements, including United States (US) 21 Code of Federal Regulations (CFR) 312.3(b) for constitution of independent ethics committees.

This study was conducted in accordance with ICH GCP and all applicable subject privacy requirements and the ethical principles that are outlined in the Declaration of Helsinki 2008. The study was monitored in accordance with ICH E6, Section 5.18. Investigators were trained to conduct the study in accordance with GCPs and the study protocol as defined in ICH E3, Section 9.6. Written commitments were obtained from investigators to comply with GCP and to conduct the study in accordance with the protocol.

Written informed consent was obtained from each subject prior to the performance of any study-specific procedures. The investigator agreed to provide the subjects as much time as necessary to review the document, to inquire about details of the trial, and to decide whether or not to participate in the study. The informed consent was signed and dated by the study subjects and by the person who conducted the informed consent discussion. Electronic case report forms were provided for each subject’s data to be recorded.

#### Study methods

Subjects who met all screening criteria were enrolled and admitted to the clinical test center 1 day before the exercise protocol. Subjects who had a creatine kinase (CK) within the normal range at baseline participated in the study. Ten minutes before the exercise protocol, range of motion, muscle pain/soreness, and MVC were measured. The exercise protocol was then conducted on the same arm (details below). Subjects who achieved a deficit from baseline of at least 40% in their MVC were randomized to either daprodustat or placebo and entered the post-exercise phase. Fifteen minutes after completion of eccentric exercise, each subject started to receive a total of five oral doses of daprodustat or placebo on day 1 (0 h, 4 h, 8 h), day 2 (24 h), and day 3 (48 h). Functional assessments performed pre-exercise were repeated at 5 min, 30 min, 24 h, 48 h, and 72 h post-exercise. Subjects were discharged from the unit on day 4 and returned for a follow-up visit 7–10 days later.

The study was performed in two separate cohorts. In cohort 1, subjects were randomized to either placebo or 5 mg daprodustat. In cohort 2, subjects were randomized to either placebo or 50 mg daprodustat. The progression to the second cohort, termination of study, or a repeat of the same dose level was based on the feasibility of the eccentric exercise protocol and the totality of the data. The data sets reviewed, at minimum, consisted of safety data and observed MVC at 90° of flexion. This study was double-blinded with respect to the subjects, the investigator and site staff (with the exception of the site pharmacist). GlaxoSmithKline (GSK) was un-blinded throughout the study. This study was performed in compliance with Good Clinical Practices and GlaxoSmithKline Standard Operating Procedures for all processes involved, including the archiving of essential documents. Anonymized individual participant data and study documents can be requested for further research from www.clinicalstudydatarequest.com.

#### Inclusion criteria

Subjects were eligible for inclusion in this study only if all of the following criteria were met:

1. Males between 18 and 35 years of age inclusive, at the time of signing the informed consent.
2. Healthy as determined by a responsible and experienced physician, based on a medical evaluation including medical history, physical examination, laboratory tests, and cardiac monitoring.
3. Body weight ≥ 70 kg and BMI within the range 22.0 to 34.0 (inclusive).
4. Capable of giving written informed consent, which included compliance with the requirements and restrictions listed in the consent form.
5. ALT, alkaline phosphatase, and bilirubin ≤ 1.5 × ULN.
6. QTc < 450 ms based on single or average QTc value of triplicate values obtained over a brief recording period.
7. At least a 1 year history of no regular (2–3 times per week) exercise and no heavy exertion within past week.
8. No strenuous exercise involving the arms during the last 10 weeks.

#### Eccentric exercise protocol

Each subject performed maximal eccentric contractions of the elbow flexors of one arm against a lever arm moving at a constant (isokinetic) angular velocity over a limited range of motion. The aim of this procedure was to elicit at least a 40% decline in isometric strength from the start to the end of the exercise. To take account of variability in performance between individuals, the exercise procedure was repeated up to three times in total to elicit this deficit. Subjects not manifesting at least a 40% decline in isometric strength were excluded from the study. Each subject will be placed on a Cybex with the lever arm fixed so that the elbow was at 90° flexion. The subject was then asked to provide an MVC of the elbow flexors by pulling against the lever arm while force production is measured. The MVC was measured three times in succession and the highest value recorded. This value was used as a reference assessment of volunteer performance in the subsequent exercise protocol. After a 2-min rest, subjects were asked to perform five sets of six maximal eccentric contractions of the elbow flexors of the non-dominant arm to induce local muscle inflammation, pain and transient functional deficits. Each eccentric contraction started at an elbow angle of 90^o^ flexion and volunteers were asked to pull against the lever arm before the lever arm extended to 180^o^ at a constant angular velocity of 60^o^/s. Subjects were verbally encouraged to maximally resist the lowering lever arm of the ergometer during the eccentric movement flexion. At the end of each eccentric contraction, each subject was instructed to relax and the lever arm returns to the 90^o^ flexion position at an angular velocity of 10^o^/s before the next eccentric contraction. Each bout of 6 contractions was separated by 2 min of resting recovery. At the end of the five sets, after a 2-min rest period, the subject was asked to provide another MVC. At this point, the instructor calculated the acute post-exercise MVC deficit. If post-exercise MVC was < 40% lower than pre-exercise, this triggered a second round of 30 eccentric contractions. If post-exercise MVC was > 40% lower than pre-exercise, the exercise was considered complete. For the subjects entering a second round of eccentric exercise, they repeated five sets of six maximal eccentric contractions with a 2-min rest period between sets as above. At the end of the five sets, after a 2-min rest period, the subjects were asked to provide an MVC. If post-exercise MVC was > 40% lower than pre-exercise, the exercise was considered complete. There was provision to go to a third round of exercise if post-exercise was < 40% of pre-exercise MVC but every subject in the study achieved a > 40% deficit within two exercise rounds.

#### Study objectives and endpoints

The primary objective of the study was to evaluate the protective effects of daprodustat on eccentric exercise-induced muscle injury by measuring MVC of the exercised arm up to 72 h after completion of eccentric exercise in subjects treated with daprodustat in comparison to subjects treated with placebo. Secondary objectives included assessment of the safety and tolerability of daprodustat by measuring adverse events, vital signs (systolic and diastolic blood pressure, pulse rate, and respiratory rate), 12-lead electrocardiogram (ECGs) (heart rate, PR, QRS, QT, and QTcF), and laboratory parameters (Additional file 6: Table S3). Another secondary objective was to evaluate the protective effects of daprodustat on deficits in arm range of motion after eccentric exercise by measuring change in degree of motion and resting arm angle from post-exercise up to 72 h after completion of eccentric exercise in subjects treated with daprodustat in comparison to subjects treated with placebo.

#### Measurement of MVC isometric strength

Strength was assessed in the non-dominant arm by a maximal isometric (fixed length) contraction. The subject was placed on a Cybex ergometer (Medway, MA) in a semi-supine position grasped the dynamometer handle with the wrist in a supinated position. MVC was measured at both 90° and 150° of extension at the elbow (90° and 30° of flexion respectively). While the subject was seated, the lever arm was fixed so that the elbow was at the appropriate angle (90° and 150° of extension/90° and 30° of flexion respectively). The subject was then asked to provide a maximal voluntary contraction of the elbow flexors by pulling against the lever arm while force-production was measured. The MVC was measured three times in succession at each angle, with a 30 s rest between attempts. The highest value at each angle was recorded.

#### Measurement of elbow range of motion (ROM)

A manual goniometer was used to measure the range of motion at the elbow. Joint angles were measured at rest, at maximum extension and at maximum flexion. Measurements were taken twice for each joint angle and the mean value of the two measurements was used to calculate the ROM by subtracting the flexion angle from the extension angle.

#### Somascan serum analysis

Baseline and 72 h serum samples from Cohort 2 were examined with an aptamer-based proteome analysis screen (SOMAlogic Inc., SOMAscan® Boulder, CO, Fig. 3d and Additional file 7: Table S4). Protein concentration data was log2 transformed and normalized using quantile normalization [44]. To determine differential expression, a linear model was fit [43] taking into account the treatment effect as well as the pair wise design (i.e., samples from the same individual before and after treatment). The false discovery rate (FDR) was computed as an adjusted *P* value to account for multiple testing and a cutoff of 10% FDR as well as an absolute fold change cutoff of 1.5 or greater was used to define differential expression. All analyses were done using the R/Bioconductor and ggplot2 software packages [46].

#### Clinical statistical analysis

Clinical primary endpoints were analyzed using a ANCOVA model with a fixed term of treatment regimen, visit, interaction of regimen and visit, baseline endpoint as a covariate and subject as a random effect. Point estimate and its 95% confidence interval were obtained from the model for comparisons of interests (e.g., active doses versus placebo at each visiting time point). Biomarkers, including serum CK activity, after logarithm transformation, were analyzed with a repeat measure ANOVA with fixed terms of regimen, visit and interaction of regimen and visit, subject as random effects.

## Additional files

Additional file 1: **Figure S1.** Prolyl hydroxylase inhibitors protect muscle at doses that raise EPO but not VEGF. (a) Effect of different daily doses of GSK360 on contraction induced injury in mice. Mice were treated with vehicle or the indicated dose of GSK360 daily. Data are normalized to pre-damage force (*n* = 8). (b) Effect of co-treatment with EPO neutralizing antibody and GSK360 for 3 days on circulating red blood cells and hematocrit (*n* = 8). All data in the figure are shown as the mean ± SEM. Two-way ANOVA followed by Holm-Sidak’s test corrected for multiple comparisons; **P < 0.01 as compared to vehicle dosed cohort. (PDF 224 kb)

Additional file 2: **Figure S2.** Prolyl hydroxylase inhibitors do not alter transcriptional profiles in skeletal muscle. (a) Transcriptional profile in mouse gastrocnemius muscle 9 h after injury with 10 mg/kg GSK360 or vehicle. After correcting for multiple testing, no statistically significant changes were detected. (b) Relative tissue concentration of GSK360 after 4 h of IV infusion (*n* = 3). (c) Flow cytometry quantification of F4/80 positive macrophages in muscle 0–14 days after muscle injury with 10 mg/kg GSK360 or vehicle (*n* = 4). (**d**) Quantification of HIF1α in polarized murine bone marrow-derived macrophages (BMDM) following 24 h of GSK360 treatment. Protein extracts from BMDMs were generated (*n* = 2). HIF1α was quantified via DuoSet ELISA (R&D Systems) with protein extracts. All data in the figure are shown as the mean ± SEM. Two-way ANOVA followed by Holm-Sidak’s test corrected for multiple comparisons; **P < 0.01 as compared to vehicle dosed cohort. (PDF 257 kb)

Additional file 3: **Table S1.** Excel file of Affymetrix Gene Chip mRNA levels from mouse gastrocnemius muscle 3, 6 and 9 hrs after injury with 10 mg/kg GSK360 vehicle. Table shows comparison of expression levels between vehicle and GSK360 treated muscle. (XLSX 1910 kb)

Additional file 4: **Figure S3.** Daprodustat does not alter arm range of motion after eccentric exercise. (a) Effect of different daily doses of daprodustat on contraction induced injury in mice. Mice were treated with vehicle or the indicated dose of daprodustat daily. Data are normalized to pre-damage force (*n* = 9). (b) Arm range of motion, measured with a manual goniometer was recorded 10 min pre-exercise and 5 min, 30 min, 24 h, 48 h, and 72 h post exercise. The boxed area represents a 75% range of values with the central line indicating the median and the cross the mean value. Individual values are displayed to the left (top panel placebo vs GSK863 5 mg (*n* = 14, *n* = 12), bottom panel placebo vs GSK863 50 mg (both *n* = 15)). (PDF 270 kb)

Additional file 5: **Table S2.** Demographic data for the two healthy volunteer studies. Twenty-six healthy volunteers were randomized in Cohort 1, 30 were randomized in cohort 2, with approximately 1:1 ratio of daprodustat vs. placebo. All randomized subjects completed the study, and the data from all randomized subjects were included in the study analyses. Overall, demographic characteristics were similar among the placebo and GSK127863 groups. No violation of the treatment assignment or broken blinding occurred in this study. No significant protocol deviations were found during the study. (PDF 200 kb)

Additional file 6: **Table S3.** Summary of adverse events. No serious adverse events (SAEs) were reported in this study. Fifteen subjects (26.8%) reported adverse events (AEs). The most commonly reported AEs (> 1 subject) were myalgia in nine subjects (16.1%), headache in two subjects (3.6%), and peripheral swelling in two subjects (3.6%). Myalgia was largely limited to arm pain during and after the exercise. Single events of vomiting and pollakiuria, both in the placebo group, were not considered by the investigator as related to study drug. No AEs led to withdrawal of subjects from this study. There were no clinically significant findings of vital signs, ECG or clinical laboratory parameters. (PDF 196 kb)

Additional file 7: **Table S4.** Effect of daprodustat on serum protein changes pre- and 3 days post exercise. Excel file for SOMAscan® (SOMAlogic Inc.) aptamer-based proteome analysis of serum comparing baseline (pre-injury) and 72 h post-injury serum samples from Cohort 2 of the healthy volunteer study. Protein concentration data was log2 transformed and normalized using quantile normalization [44]. To determine differential expression, a linear model was fit [43] taking into account the treatment effect as well as the pair wise design (i.e., samples from the same individual before and after treatment). (PDF 1428 kb)

## Abbreviations

BMDM: Bone marrow-derived macrophage
EPO: Erythropoietin
HIF: Hypoxia-induced factor
MVC: Maximum voluntary contraction
NOS: Nitric oxide synthase
PHD: Prolyl hydroxylase
PHI: Prolyl hydroxylase inhibitor
