# Correction to: HIF prolyl hydroxylase inhibition protects skeletal muscle from eccentric contraction induced injury

**DOI:** 10.1186/s13395-018-0185-7

**Published:** 2018-12-10

**Authors:** Andrew N. Billin, Samuel E. Honeycutt, Alan V. McDougal, Jaclyn P. Kerr, Zhe Chen, Johannes M. Freudenberg, Deepak K. Rajpal, Guizhen Luo, Henning Fritz Kramer, Robert S. Geske, Frank Fang, Bert Yao, Richard V. Clark, John Lepore, Alex Cobitz, Ram Miller, Kazunori Nosaka, Aaron C. Hinken, Alan J. Russell

**Affiliations:** 1 Muscle Metabolism Discovery Performance Unit, GlaxoSmithKline, King of Prussia, PA USA; 2 Metabolic Pathways and Cardiovascular Therapy Area, GlaxoSmithKline, King of Prussia, PA USA; 3 Target Sciences, GlaxoSmithKline, King of Prussia, PA USA; 4 Clinical Statistics, GlaxoSmithKline, King of Prussia, PA USA; 5 School of Medical and Health Sciences, Edith Cowan University, Joondalup, WA Australia

**Correction to: Skeletal Muscle (2018) 8:35**

**https://doi.org/10.1186/s13395-018-0179-5**

Following publication of the original article [1], the authors flagged that there is a discrepancy with the *Availability of data and materials* statement on page 12 of the article.

This statement says that “Raw preclinical and clinical data for this manuscript will not be shared for logistical reasons.”

However, the authors state on page 10 of the article that “Anonymized individual participant data and study documents can be requested for further research from www.clinicalstudydatarequest.com.”

The policy at GlaxoSmithKline, Inc., the sponsor of this paper (see the *Funding* section on page 12), is consistent with the latter statement, of page 10.

As such, in the interest of full transparency, please be advised that **the** ***Availability of data and materials*** **statement on page 12 of the article is void**.
