# Supplementary figures and images for: HIF prolyl hydroxylase inhibition protects skeletal muscle from eccentric contraction-induced injury
Source: Skelet Muscle. 2018 Nov 13;8:35. doi: 10.1186/s13395-018-0179-5 (PMC6234580; doi:10.1186/s13395-018-0179-5)

Figure S1

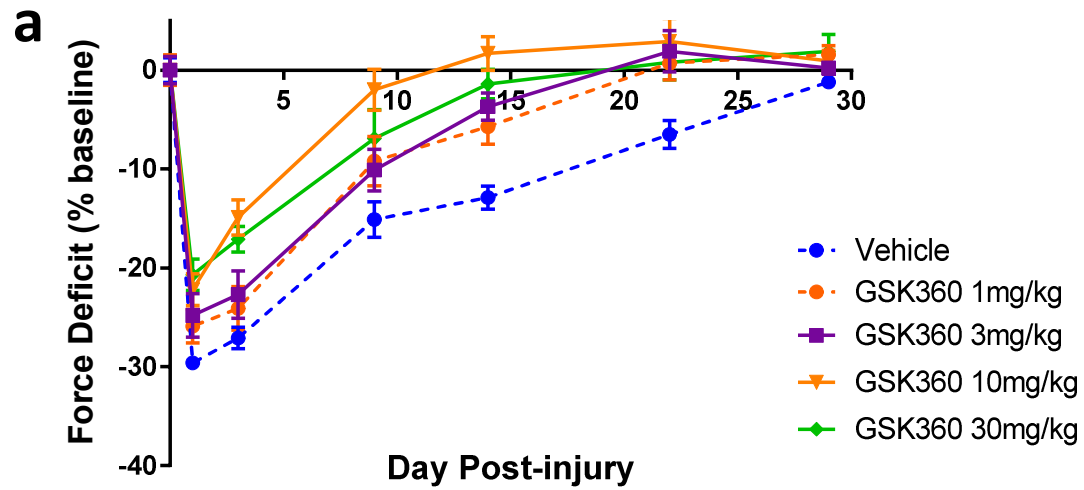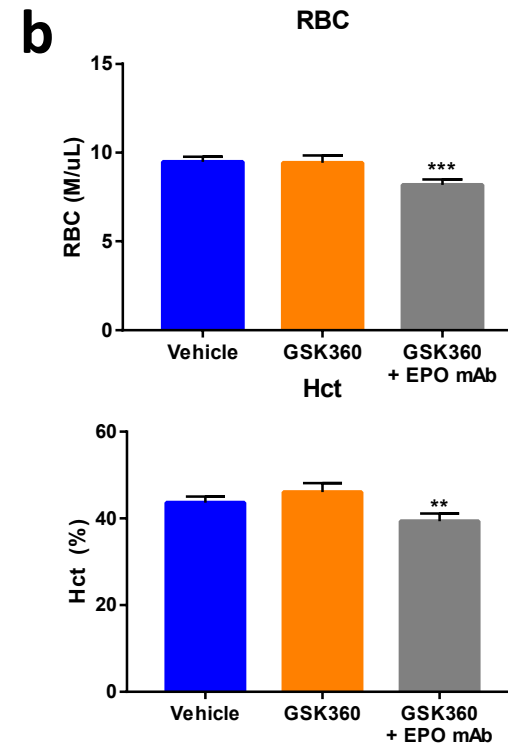

Supplement: Supplementary file 1 — Figure S1. Prolyl hydroxylase inhibitors protect muscle at doses that raise EPO but not VEGF. (a) Effect of different daily doses of GSK360 on contraction induced injury in mice. Mice were treated with vehicle or the indicated dose of GSK360 daily. Data are normalized to pre-damage force (n = 8). (b) Effect of co-treatment with EPO neutralizing antibody and GSK360 for 3 days on circulating red blood cells and hematocrit (n = 8). All data in the figure are shown as the mean ± SEM. Two-way ANOVA followed by Holm-Sidak’s test corrected for multiple comparisons; **P < 0.01 as compared to vehicle dosed cohort. (PDF 224 kb) [file 13395_2018_179_MOESM1_ESM.pdf]

# Figure S2

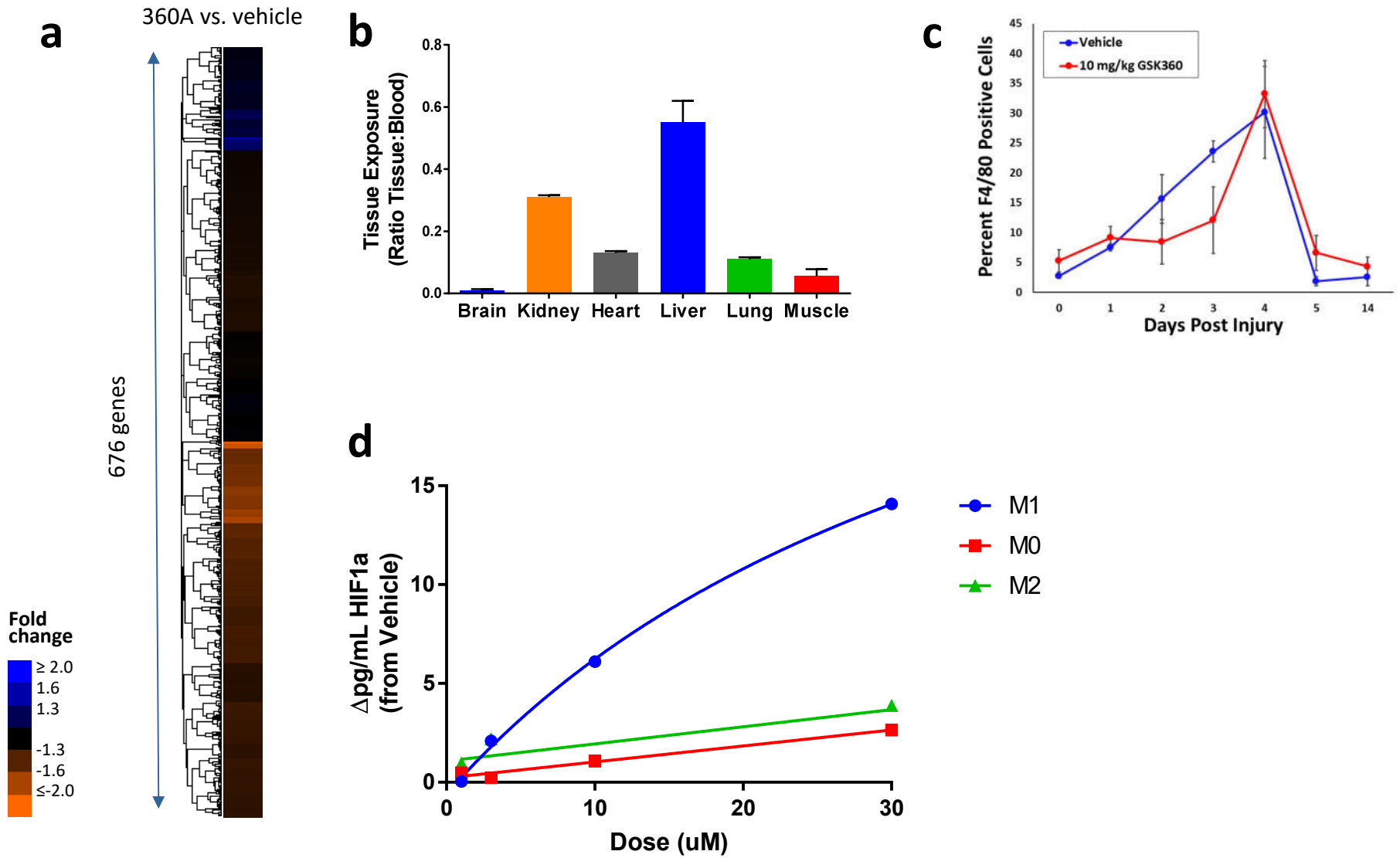

Supplement: Supplementary file 2 — Figure S2. Prolyl hydroxylase inhibitors do not alter transcriptional profiles in skeletal muscle. (a) Transcriptional profile in mouse gastrocnemius muscle 9 h after injury with 10 mg/kg GSK360 or vehicle. After correcting for multiple testing, no statistically significant changes were detected. (b) Relative tissue concentration of GSK360 after 4 h of IV infusion (n = 3). (c) Flow cytometry quantification of F4/80 positive macrophages in muscle 0–14 days after muscle injury with 10 mg/kg GSK360 or vehicle (n = 4). (d) Quantification of HIF1α in polarized murine bone marrow-derived macrophages (BMDM) following 24 h of GSK360 treatment. Protein extracts from BMDMs were generated (n = 2). HIF1α was quantified via DuoSet ELISA (R&D Systems) with protein extracts. All data in the figure are shown as the mean ± SEM. Two-way ANOVA followed by Holm-Sidak’s test corrected for multiple comparisons; **P < 0.01 as compared to vehicle dosed cohort. (PDF 257 kb) [file 13395_2018_179_MOESM2_ESM.pdf]

# Figure S3

**a**

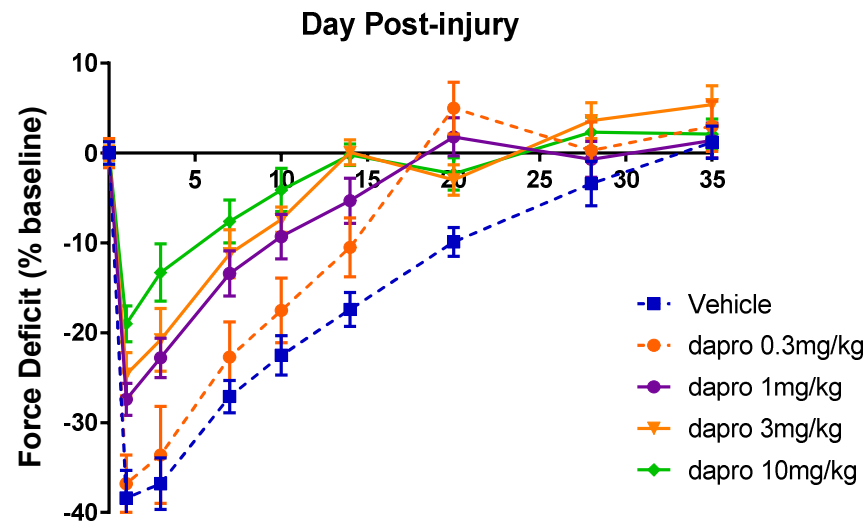

**b**

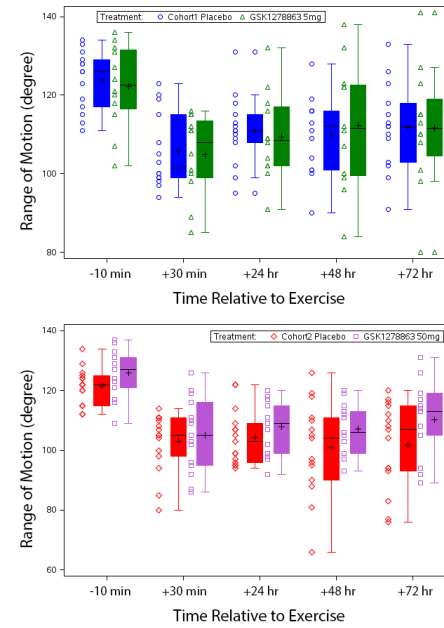

Supplement: Supplementary file 4 — Figure S3. Daprodustat does not alter arm range of motion after eccentric exercise. (a) Effect of different daily doses of daprodustat on contraction induced injury in mice. Mice were treated with vehicle or the indicated dose of daprodustat daily. Data are normalized to pre-damage force (n = 9). (b) Arm range of motion, measured with a manual goniometer was recorded 10 min pre-exercise and 5 min, 30 min, 24 h, 48 h, and 72 h post exercise. The boxed area represents a 75% range of values with the central line indicating the median and the cross the mean value. Individual values are displayed to the left (top panel placebo vs GSK863 5 mg (n = 14, n = 12), bottom panel placebo vs GSK863 50 mg (both n = 15)). (PDF 270 kb) [file 13395_2018_179_MOESM4_ESM.pdf]
