# Supplementary material for: HIF prolyl hydroxylase inhibition protects skeletal muscle from eccentric contraction-induced injury
Source: Skelet Muscle. 2018 Nov 13;8:35. doi: 10.1186/s13395-018-0179-5 (PMC6234580; doi:10.1186/s13395-018-0179-5)
Supplement: Supplementary file 5 — Table S2. Demographic data for the two healthy volunteer studies. Twenty-six healthy volunteers were randomized in Cohort 1, 30 were randomized in cohort 2, with approximately 1:1 ratio of daprodustat vs. placebo. All randomized subjects completed the study, and the data from all randomized subjects were included in the study analyses. Overall, demographic characteristics were similar among the placebo and GSK127863 groups. No violation of the treatment assignment or broken blinding occurred in this study. No significant protocol deviations were found during the study. (PDF 200 kb) [file 13395_2018_179_MOESM5_ESM.pdf]

Table S2

**Number of Subjects**	**Cohort 1 Placebo**	**Cohort 1 GSK1278863 5 mg**	**Cohort 2 Placebo**	**Cohort 2 GSK1278863 50 mg**
**Number of subjects** [N]	14	12	15	15
**Age in Years** [Mean (SD)]	28.2 (3.36)	29.3 (3.70)	29.4 (4.87)	28.2 (4.51)
**Sex** [n]				
Female	0	0	0	0
Male	14	12	15	15
**BMI** (kg/m^2) [Mean (SD)]	27.3 (2.8)	26.8 (3.3)	28.5 (3.4)	26.0 (3.0)
**Height** (cm) [Mean (SD)]	177.1 (6.2)	179.8 (7.5)	179.8 (6.2)	180.5 (6.7)
**Weight** (kg) [Mean (SD)]	85.4 (7.8)	86.4 (10.4)	92.2 (12.3)	85.0 (13.5)
**Ethnicity** [n (%)]				
Hispanic or Latino	2 (14%)	2 (17%)	2 (13%)	1 (7%)
Not Hispanic or Latino	12 (86%)	10 (83%)	13 (87%)	14 (93%)
**Race** [n (%)]				
African American/African Heritage	12 (86%)	10 (83%)	9(60%)	12 (80%)
White	2 (14%)	2 (17%)	6 (40%)	3 (20%)
