# Supplementary material for: HIF prolyl hydroxylase inhibition protects skeletal muscle from eccentric contraction-induced injury
Source: Skelet Muscle. 2018 Nov 13;8:35. doi: 10.1186/s13395-018-0179-5 (PMC6234580; doi:10.1186/s13395-018-0179-5)
Supplement: Supplementary file 6 — Table S3. Summary of adverse events. No serious adverse events (SAEs) were reported in this study. Fifteen subjects (26.8%) reported adverse events (AEs). The most commonly reported AEs (> 1 subject) were myalgia in nine subjects (16.1%), headache in two subjects (3.6%), and peripheral swelling in two subjects (3.6%). Myalgia was largely limited to arm pain during and after the exercise. Single events of vomiting and pollakiuria, both in the placebo group, were not considered by the investigator as related to study drug. No AEs led to withdrawal of subjects from this study. There were no clinically significant findings of vital signs, ECG or clinical laboratory parameters. (PDF 196 kb) [file 13395_2018_179_MOESM6_ESM.pdf]

Table S3

Adverse Events	Cohort 1 Placebo	GSK1278863 5 mg	Cohort 2 Placebo	GSK1278863 50 mg
	N=14	N=12	N=15	N=15
	n (%)	n (%)	n (%)	n (%)
Any AE	4 (29%)	6 (50%)	3 (20%)	2 (13%)
Any AE related to investigational product	0	0	2 (13%)	0
All AEs:				
Myalgia	3 (21%)	4 (33%)	1 (7%)	1 (7%)
Headache	0	0	1 (7%)	1 (7%)
Peripheral swelling	0	1 (8%)	1 (7%)	0
Arthralgia	0	1 (8%)	0	0
Fatigue	1 (7%)	0	0	0
Oedema peripheral	0	0	1 (7%)	0
Somnolence	1 (7%)	0	0	0
Mouth ulceration	0	1 (8%)	0	0
Vomiting	0	0	1 (7%)*	0
Acne	0	0	1 (7%)	0
Dermatitis contact	0	1 (8%)	0	0
Pollakiuria	0	0	1 (7%)*	0

Note: * considered by the investigator to be related to study medication
